# The Test of Time

**Published:** 1912-10

**Authors:** Oscar F. Baerens

**Affiliations:** Professor of Diseases of the Ear, Nose and Throat, St. Louis College of Physicians and Surgeons, St. Louis, Mo.


### Abstracts and Selections.

### The Test of Time.

BY OSCAR F. BAERENS, M. D., PH. G.,

Professor of Diseases of the Ear, Nose and Throat, St. Louis College of Physicians and Surgeons, St. Louis, Mo.

When a medical student has learned the fundamental principles of medicine and surgery and assimilated them, he is usually a safe man to send to the bedside with a pencil and pad of blanks. These fundamentals are the fruits from the tree of experience, and constitute the physician's most valuable possessions.

As a teacher, I have learned that maxims and aphorisms impress themselves upon the minds of my students more readily than a lengthy discourse, and it has always been my custom to interpret them in my discourse and clinical lectures on diseases of the ear, nose and throat.

Limiting, as I do, my practice to these diseases, I, as a matter of course, am more familiar with the requirements and needs of patients of this class, and I have learned that cleanliness in addition to being next to godliness constitutes about 60 per cent of the treatment of simple catarrhal conditions of these structures. In this era of rapid-fire pharmacy the doctor is almost daily the recipient of samples and literature pertaining to them, and much of his valuable time is consumed by the detail men and representatives of manufacturing pharmacists. While it is not denied that hosts of these pharmacals are meritorious to a greater or less degree, it can neither be argued that the average life is too short to carry on a series of laboratory and clinical experiments to verify the statements of their producers. I believe it to be most profitable to thoroughly familiarize one's self with a given number of standard preparations and then to use them whenever and wherever indicated. During my experience as a pharmacist I have had ample opportunity to learn the merits of properly prepared pharmacals, and I believe that I know one of these when I see one. In Glyco-Thymoline I have found a preparation upon which the body medical has placed the seal of approval and one calculated to meet the requirements of the medical practician's varied needs. The preparation is too well known by reason of its worldwide (I use this term advisedly) use to necessitate or warrant a description or analysis here, nor do I propose to speak for others beside myself. It is, however, a pleasure to state that for the past eight years I have used this preparation to the exclusion of all others in my work at the clinic and in private practice whenever I wanted a mild cleansing antiseptic detergent remedy.

---

Our attention has been called to the fact that the health plank quoted from the platform of the National Progressive party, in our issue of last week, is not the only or most important utterance of the party on the subject of the physical well-being of the people. We, therefore, give the following plank, which the leaders of the party claim should receive the earnest attention of medical men. The full plank is as follows:

"The supreme duty of the nation is the conservation of human resources through an enlarged measure of social and industrial justice. We pledge ourselves to work unceasingly in State and nation for:

"Effective legislation looking to the prevention of industrial accidents, occupational diseases, overwork, involuntary employment and other injurious effects incident to modern industry.

"The fixing of minimum safety and health standards for the various occupations and the exercise of the public authority of State and nation, including the federal control over interstate commerce, and the taxing power to maintain such standards.

"The prohibition of child labor.

"Minimum wage standards for working women, to provide a 'living wage' in all industrial occupations.

"The general prohibition of night work for women and the establishment of an eight-hour day for women and young persons.

"One day's rest in seven for all wageworkers.

"The eight-hour day in continuous twenty-four-hour industries.

"The abolition of the contract labor system; substituting a system of prison production for governmental consumption only, and the application of prisoners' earnings to the support of their dependent families.

"Publicity as to wages, hours and conditions of labor.

"Full reports upon industrial accidents and diseases, and the opening to public inspection of all tallies, weights, measures and check systems on labor products."—*Lancet-Clinic.*

